# Surgical management of urolithiasis – a systematic analysis of available guidelines

**DOI:** 10.1186/s12894-018-0332-9

**Published:** 2018-04-10

**Authors:** Valentin Zumstein, Patrick Betschart, Dominik Abt, Hans-Peter Schmid, Cedric Michael Panje, Paul Martin Putora

**Affiliations:** 1 Department of Urology, Cantonal Hospital St. Gallen, St. Gallen, Switzerland; 2 Department of Urology, University Medical Center Hamburg-Eppendorf, Hamburg, Germany; 3 Department of Radiation Oncology, Cantonal Hospital St. Gallen, St. Gallen, Switzerland; 4 Department of Radiation Oncology, lnselspital, Bern University Hospital, Bern, Switzerland

**Keywords:** Consensus, Guidelines, Urolithiasis, Management, Surgical, Decision tree

## Abstract

**Background:**

Several societies around the world issue guidelines incorporating the latest evidence. However, even the most commonly cited guidelines of the European Association of Urology (EAU) and the American Urological Association (AUA) leave the clinician with several treatment options and differ on specific points. We aimed to identify discrepancies and areas of consensus between guidelines to give novel insights into areas where low consensus between the guideline panels exists, and therefore where more evidence might increase consensus.

**Methods:**

The webpages of the 61 members of the Societé Internationale d’Urologie were analysed to identify all listed or linked guidelines. Decision trees for the surgical management of urolithiasis were derived, and a comparative analysis was performed to determine consensus and discrepancies.

**Results:**

Five national and one international guideline (EAU) on surgical stone treatment were available for analysis. While 7 national urological societies refer to the AUA guidelines and 11 to the EAU guidelines, 43 neither publish their own guidelines nor refer to others. Comparative analysis revealed a high degree of consensus for most renal and ureteral stone scenarios. Nevertheless, we also identified a variety of discrepancies between the different guidelines, the largest being the approach to the treatment of proximal ureteral calculi and larger renal calculi.

**Conclusions:**

Six guidelines with recommendations for the surgical treatment of urolithiasis to support urologists in decision-making were available for inclusion in our analysis. While there is a high grade of consensus for most stone scenarios, we also detected some discrepancies between different guidelines. These are, however, controversial situations where adequate evidence to assist with decision-making has yet to be elicited by further research.

## Background

A range of procedures is in use for the surgical treatment of urolithiasis. Treatment strategies are mainly based on stone location and size, and the patient’s comorbidities and preferences. Guidelines have been developed to support clinicians in selecting the most appropriate treatment in controversial situations. Several institutions around the world have issued guidelines incorporating the latest evidence.

However, even the most commonly cited guidelines of the European Association of Urology (EAU) and the American Urological Association (AUA) leave the clinician with several treatment options and differ on specific points, such as cut-off values for stone size and recommendations for the treatment of choice [1–3]. Ambiguities and discrepancies between different guidelines may result from different interpretations of the evidence available and possible methodological differences in guideline creation. Therefore, careful analysis of the similarities and differences between different sources can provide additional insight [4].

We aimed to determine how many guidelines on the surgical management of urolithiasis actually exist and the urological associations that recommended them. We systematically analysed the criteria proposed for decision-making and the recommended surgical approaches in each guideline. In addition, we aimed to identify discrepancies and areas of consensus between guidelines, with particular attention to the two major guidelines, those of the EAU and AUA.

This work provides a systematic analysis of the recommended surgical management of urolithiasis worldwide, and gives novel insights into areas where low consensus between the guideline panels exists, and therefore where more evidence might increase consensus.

## Methods

Guidelines were selected using the membership list of the Societé Internationale d’Urologie (SIU) (http://www.siu-urology.org/society/national-delegates). The webpages of all 61 members that are represented by delegates were analysed for mentions of and links to guidelines for the surgical management of renal and ureteral calculi.

Two authors (V.Z., P.B.) independently assessed all guidelines, and decision trees for the surgical management of urolithiasis were derived, followed by crosschecking and clarification of any differences by a third author (D.A.). The methodology of this approach has been recently described [5] and was successfully used in different fields including radiotherapy in prostate cancer [6], expert opinions of the systemic treatment of recurrent glioblastoma [7], renal cell carcinoma [8, 9] and sarcoma [10]. In cases where first-, second- and or even third-line recommendations were provided, all treatment options were included into the decision trees regardless of their hierarchical level. Although hierarchical levels were assessed, they were not incorporated into the decision trees. Decision trees were built based on the criteria used in the guidelines analysed. These were stone location, i.e. renal non-lower pole, renal lower pole, and proximal and distal ureter; and stone size, i.e. > 20 mm, 10–20 mm, < 10 mm for renal stones, and > 10 mm or < 10 mm for ureteral stones [1, 2]. All treatment modalities mentioned in the different guidelines were included in our analyses: shock wave lithotripsy (SWL), percutaneous nephrolithotomy (PNL/PCNL), ureterorenoscopy including flexible and semi-rigid URS, covering also the terms retrograde intrarenal surgery (RIRS) and cirurgia intrarenal retrograda (CIRR) as described in the EAU and Sociedad Argentina de Urologia (SAU) guidelines [2, 11, 12], and open surgery. Patient preference and contraindications were considered to be universal factors and were therefore omitted from the analysis. Moreover, recommendations on conservative treatment, special cases (e.g. stone management in pregnancy, staghorn stones, cysteine stones) and postoperative follow-up were not part of our systematic analysis.

All decision trees were analysed and compared to each other to determine consensus or discrepancies between each possible combination of parameters using web-based software (Diagnostic Nodes), as described previously [5–7, 13].

To evaluate discrepancies, a combined tree containing all recommendations was generated. A mode tree was also generated to identify the most common combination of recommendations for each possible situation.

Consensus was defined as complete overlap between the recommended treatments for any case. If all guidelines recommended only one therapy for a specific situation, agreement was 100%. However, if three of six guidelines recommended therapy A only, and the others therapy A or B, this resulted in only 50% agreement for therapy A.

In addition to comparing all guidelines, the two international and most frequently cited guidelines issued by the EAU and AUA were separately compared to highlight discrepancies or areas of consensus.

## Results

Analysis of the websites of the 61 member associations represented by delegates of the SIU showed 6 national guidelines: AUA – American Urological Association [1, 3], SAU – Sociedad Argentina de Urologia [11, 12]; AFU – French Association of Urology [14]; DGU – German Society for Urology [15]; and SUA – Singapore Urological Association [16]; and the international guidelines from the EAU [2]. Some national guidelines (e.g. guideline of the Japanese Urology Association) were not included into the analysis due to linguistic difficulties caused by font systems or scripts. Eleven national urological societies refer website users to the EAU guidelines or the AUA guidelines (5 to both the EAU and AUA), 43 did not publish their own guidelines or refer readers to any others (Table 1).

**Table 1:** Recommendations of the SIU members represented by delegates regarding surgical stone treatment

SIU Member	Own guideline	Reference to other guideline	Language / latest version
Albania	No	No	
Argentina	Yes		Spanish / 2014
Australia	No	EAU	
Austria	No	EAU / AUA	
Brazil	No	No	
Canada	No^a^	No	
China	No	No	
Colombia	No^b^	No	
Costa Rica	No	No	
Cuba	No	No	
Cyprus	No	No	
Czech Rep.	No	EAU	
Egypt	No	EAU / AUA	
Finland	No	No	
France	Yes		French / 2004
Germany	Yes		German / 2016
Ghana	No	No	
Greece	No	No	
Guyana	No	No	
Haiti	No	No	
Hungary	No	No	
India	No	No	
Indonesia	No	No	
Iran	No	No	
Israel	No	No	
Italy	No	No	
Jamaica	No	No	
Japan	Yes		Japanese
Jordan	No	EAU / AUA	
Kenya	No	No	
Latvia	No	No	
Liberia	No	No	
Libya	No	No	
Lithuania	No	No	
Malaysia	No	EAU	
Mauritius	No	No	
Morocco	No	No	
Myanmar	No	No	
Netherlands	No	EAU	
Nigeria	No	No	
Norway	No	No	
Pakistan	No	No	
Palestine	No	No	
Peru	No	No	
Portugal	No	EAU	
Puerto Rico	No	AUA	
Romania	No	No	
Russia	No	No	
Serbia	No	No	
Singapore	Yes		English / 2001
Slovakia	No	No	
South Africa	No	EAU/AUA	
South Korea	No	No	
Sudan	No	No	
Sweden	No	No	
Switzerland	No	EAU / AUA	
Turkey	No	No	
Ukraine	No	No	
United Kingdom	No^c^	EAU	
United States	Yes	AUA	
Zimbabwe	No	No	

^a^No guidelines for surgical management of renal calculi

^b^No guidelines for surgical management of ureteral calculi

^c^Guidelines for renal and ureteric stones in development (anticipated publication Feburary 2019)

Decision trees were able to be derived from all guidelines identified. The site of the stone is classified consistently, i.e. proximal or distal ureteral, and lower pole or non-lower pole renal calculi, in all guidelines, except the AFU guidelines, which do not explicitly classify lower pole renal stones as an entity in their own right.

While most guidelines distinguish between > 10 mm and < 10 mm for ureteral stones, stone size is not specifically mentioned for distal ureteral calculi in the DGU recommendations or for proximal ureteral calculi in the AFU guidelines.

With regard to renal stone size, thresholds of < 10 mm, 10–20 mm and > 20 mm are used in the EAU, DGU and SAU guidelines, whilst the AUA guidelines differentiate between lower pole calculi > 10 mm and ≤ 10 mm, and non-lower pole stones > 20 mm and ≤ 20 mm. The SUA guidelines provide recommendations for lower pole calculi regardless of size, and the AFU guidelines differentiate between > 20 mm and < 20 mm for all renal stones.

**Fig. 1:**
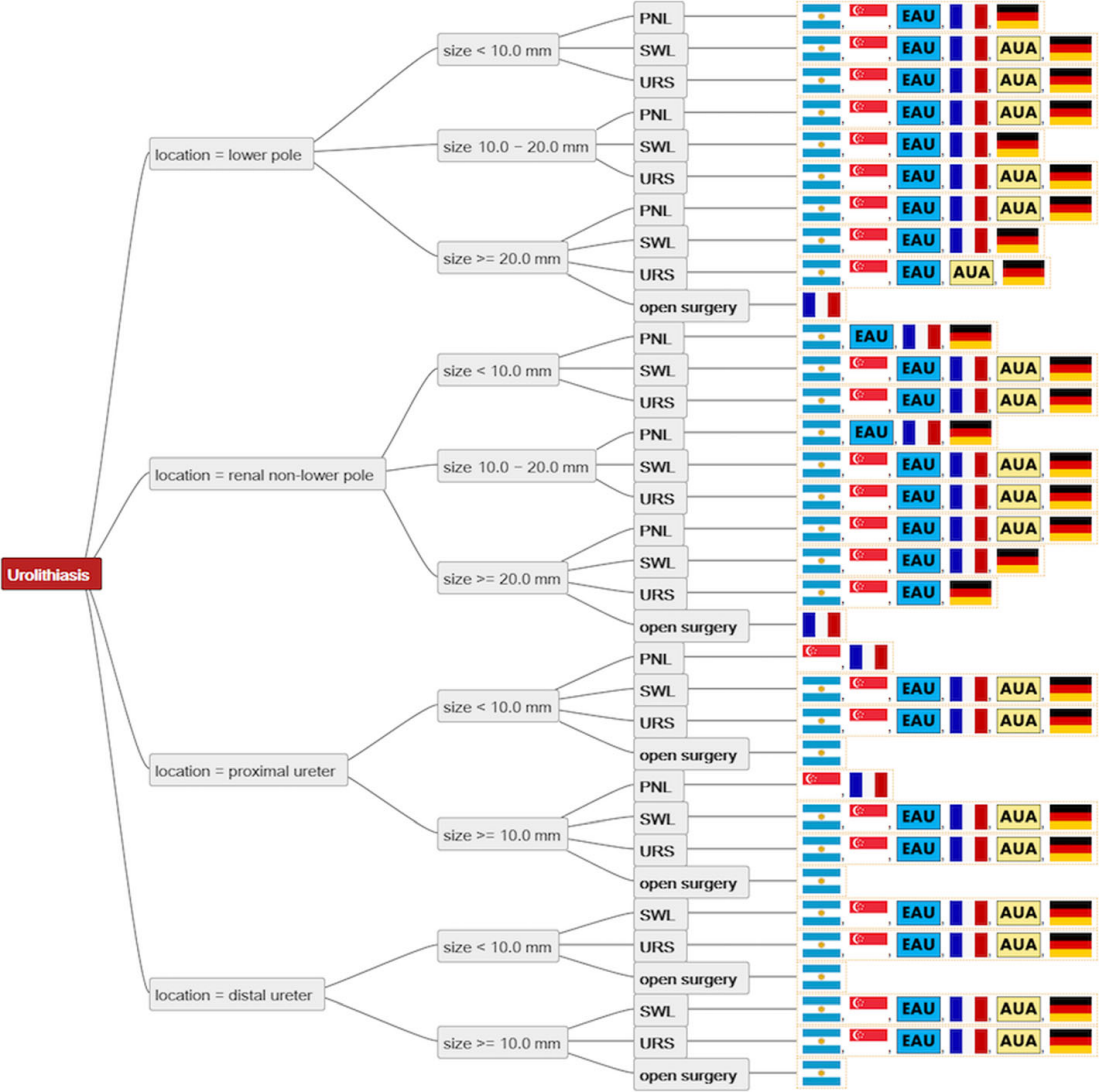
Consensus tree listing decision criteria and recommended treatments of all guidelines. (Note that URS for proximal ureteral calculi > 10 mm involves ante- and retrograde approach in the EAU-Guidelines)

Figure 1 shows the consensus decision tree resulting from semi-automatic comparison of all decision trees. SWL and URS were the most commonly recommended procedures for all stone sizes and locations. Moreover, PNL is mentioned as a treatment option for all renal calculi by all guidelines except for the AUA and SUA guidelines, which do not recommend it for the treatment of smaller non-lower pole renal calculi.

**Fig. 2:**
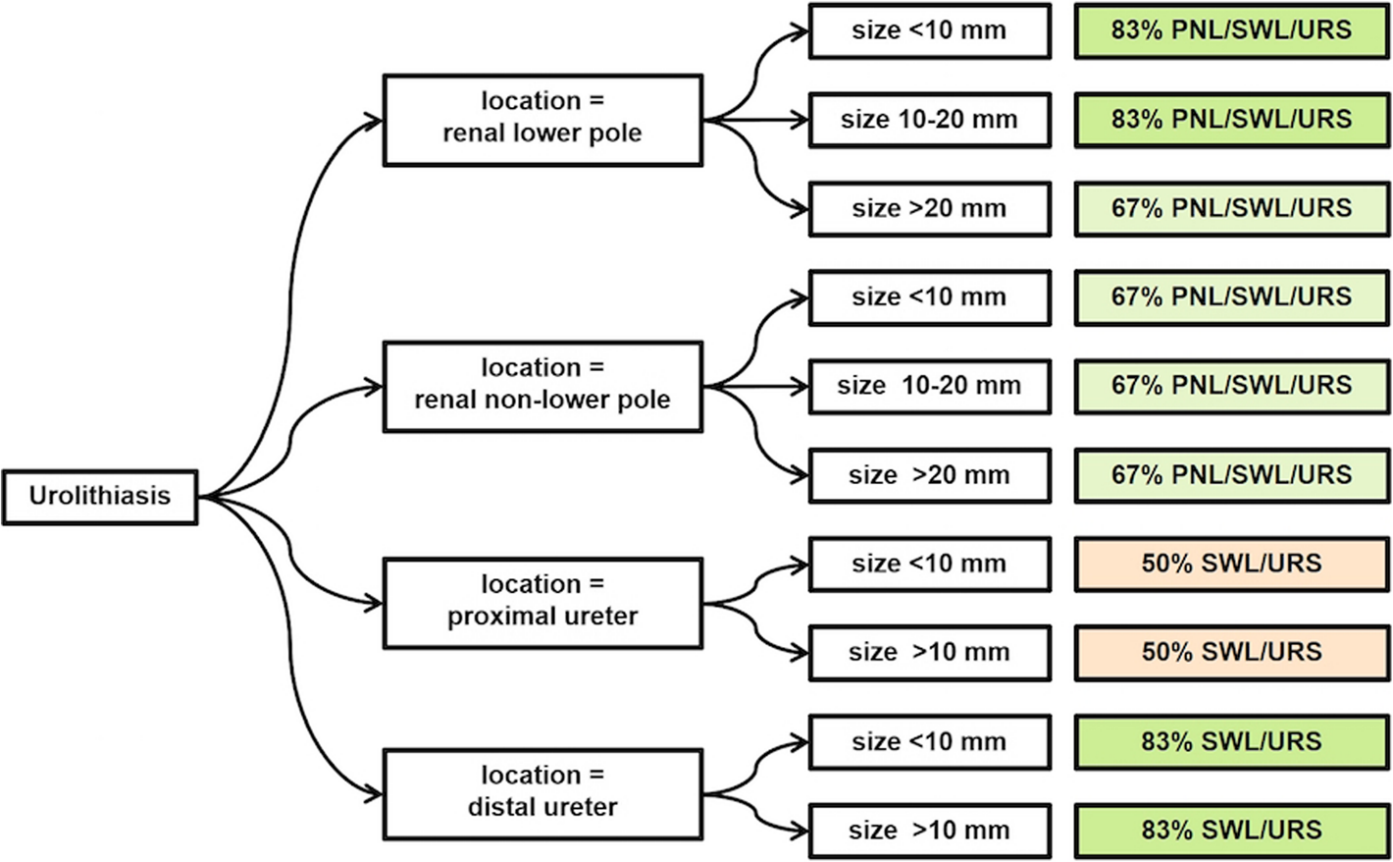
Mode tree listing the degree of agreement for the most often recommended therapeutic options. (Note that URS for proximal ureteral calculi > 10 mm involves ante- and retrograde approach in the EAU-Guidelines)

Comparative analysis assessing the most often recommended combination of procedures revealed an agreement ranging from 50 to 83% for different stone sizes and locations (Fig. 2). A high degree of consensus was found in particular for lower pole renal stones below 20 mm (83% of the guidelines recommend ‘SWL or URS or PNL’) and distal ureteral stones (83% of the guidelines recommend ‘SWL or URS’).

In contrast, we saw a low level of agreement for the treatment of proximal ureteral stones. The EAU, AUA and DGU guidelines recommend SWL or URS, whereas the other 3 guidelines additionally list PNL and open surgery, resulting in a 50% consensus for ‘SWL or URS’ as the most common recommendation.

An intermediate level of agreement was found for the remaining situations (67% consensus for renal non-lower pole calculi and lower pole calculi > 20 mm).

**Fig. 3:**
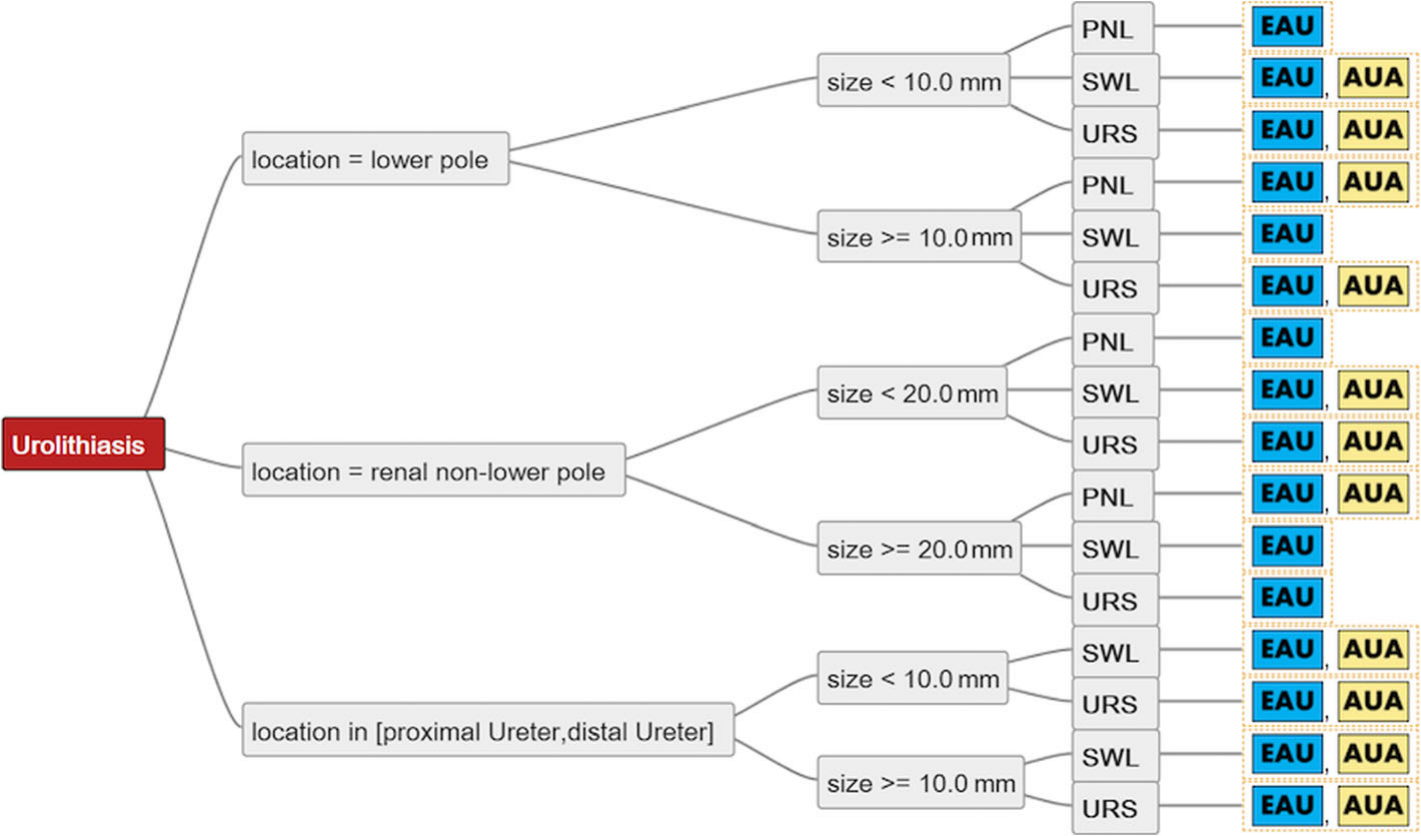
Comparison of the decision trees of EAU and AUA guidelines. Note that URS for proximal ureteral calculi > 10 mm involves ante- and retrograde approach in EAU-Guidelines

A separate comparison of the EAU and AUA guidelines including all recommended treatment options regardless of hierarchical level showed complete consensus for the treatment of ureteral calculi (Fig. 3). However, the EAU guidelines provide wider scope for decision-making regarding the treatment of renal stones, especially for larger non-lower pole calculi, and the use of PNL.

All guidelines provide a hierarchical listing of the recommended therapies for each situation (Table 2). The best agreement between the hierarchical recommendations in the six guidelines was found for distal ureteral calculi.

**Table 2:**
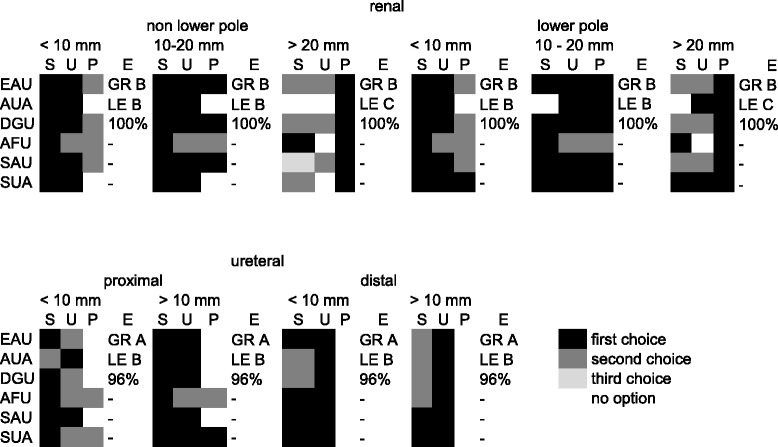
Hierarchical levels of the most commonly recommended therapies (SWL, URS, PNL) in different guidelines

*S* SWL, *U* URS, *P* PNL, *E* Evidence declaration with GR Grade of recommendation in EAU, *LE* Level of evidence in AUA, Degree of Panel consensus in DGU

SWL is recommended as first line therapy in all guidelines for smaller non-lower pole renal stones (< 20 mm), whilst only the AFU guidelines recommend SWL as first choice for > 20 mm calculi. The situation for PNL in non-lower pole and lower pole calculi is different: for small calculi < 10 mm, PNL is first choice in only the SUA guidelines, whereas for larger renal calculi > 20 mm, PNL is listed in all guidelines as first-line therapy.

Regarding proximal ureteral calculi < 10 mm, SWL is first-line therapy in all guidelines except those of the AUA, where URS is recommended as first line and SWL as second line. PNL is not recommended in this situation except for by the AFU and SUA guidelines.

URS is recommended as first line for all distal ureteral calculi, regardless of size.

## Discussion

If surgical treatment of ureteral or renal stones is indicated, clinicians face the challenge of choosing the most appropriate treatment for each patient. In ambiguous situations, evidence-based guidelines can help the urologist with decision-making.

Our systematic search covering all members of the SIU showed that the EAU and AUA guidelines are the most frequently referenced guidelines worldwide for the treatment of urolithiasis. Treatment recommendations are based on stone size and location in all available guidelines. Remarkably, the websites of most national urological associations neither refer to a reference guideline nor provide their own guidelines. However, efforts are made to improve this situation such as those by the British Association of Urological Surgeons (BAUS) in collaboration with the National Institute for Health and Care Excellence (NICE). Besides a linked guideline on laparoscopic stone removal, new guidelines covering the management of renal and ureteric calculi are supposed to be published by February 2019 (https://www.nice.org.uk/guidance/conditions-and-diseases/kidney-conditions/renal-stones). We suggest that urological associations without own guidelines should refer to one of the cited guidelines to provide a reliable source their members can refer to.

Regarding location – except for the AFU guidelines [14] – all guidelines consider calculi in the lower renal pole separately because of a reduced possibility of passage of fragments [15, 17–20]. Most guidelines categorize stone size into < 10 mm, 10–20 mm, and > 20 mm. However, the AUA guidelines for renal calculi only differentiate between ≤10 mm and > 10 mm for lower-pole stones and between ≤20 mm and > 20 mm for non-lower pole stones [1, 3]. There is also some deviation from the most common classifications in the AFU guidelines [14], SAU guidelines [11, 12], and SUA guidelines [16].

Surgical treatment of ureteral calculi depends on stone location and size. The AUA guidelines state that earlier classifications split the ureter into thirds and that this was because of the surgical approaches available. Nowadays, the ureter is divided into two sections marked by the crossing of the iliac vessels. All guidelines use a cut-off level of 10 mm to define the surgical approach.

Concerning hierarchical recommendations, all guidelines give treatment options in multiple scenarios, listed as equal or, in some cases, as first-, second- or third-line surgical treatment. To prevent the loss of recommended second- or third-line surgical therapies in our comparative analysis, we included all surgical procedures proposed as “standard procedures”, regardless of their hierarchical position in the guideline text.

Our consensus tree showed a high degree of consensus for most recommended procedures in nearly all ureteral and renal stone scenarios. We did, however, detect some significant differences, mainly concerned with the rating of SWL, where little consensus between guidelines for larger renal, distal ureteral and small proximal ureteral calculi was found. One reason for that might be geographical discrepancies in the technical performance of interventions, such as the strict use of X-ray-localisation systems for SWL in the United States compared to the widely available ultrasound guidance in Europe. Considering the rapid technological improvements of URS and PNL, further evidence seems to be required here.

While the mode tree showed a high degree of agreement for lower pole renal stones of < 10 mm and 10–20 mm and distal ureteral stones, a low level of agreement was found for proximal ureteral stones. This can be explained by the AFU and SUA guidelines also recommending PNL for proximal ureteral stones, and the SAU guidelines also recommending open surgery. However, the reason for this might be rather missing updates of some guidelines in the recent past, than a real lack of evidence for these scenarios. Thus, the last revisions of the SUA [16] and AFU guidelines [14] were issued in 2001 and 2004 and do not, therefore, reflect the latest developments.

All other guidelines clearly focus on SWL and URS in such cases. However, at this point it must be mentioned, that EAU guidelines consider a percutaneous approach for proximal ureteral calculi under the term “antegrade URS” and state that percutaneous antegrade removal of ureteral stones should be considered in selected cases [2].

The comparison of the EAU and AUA guidelines as the most commonly cited guidelines worldwide revealed several discrepancies. In general, the EAU guidelines give more therapeutic options for specific situations, delegating the choice of the appropriate treatment to the urologist and patient’s preference.

Since several approaches may be appropriate in specific situations, guidelines also rate procedures hierarchically in such cases. Our analysis of these hierarchical listings revealed a number of discrepancies between guidelines, showing that for the choice between SWL and URS, available data might have been interpreted differently. While the AUA guidelines refer to an unpublished systematic review conducted by the guidelines-panel [1], the EAU guidelines recommendation is based on a meta-analysis [18] and a work carried out by Hong et al. [19].

Moreover, PNL is mentioned as a treatment option for all renal calculi by all guidelines, except for the AUA and SUA guidelines, which do not recommend it for the treatment of smaller non-lower pole renal calculi. Although recent developments in minimal invasive PNL techniques are mentioned in the EAU and AUA guidelines, these procedures are not yet listed separately in the recommendations. The comparison of recent advances in PNL is particularly difficult because different approaches are associated with substantially different degrees of invasiveness and technical complexity.

SWL still plays an important role in all guidelines. This is remarkable because several recent studies have shown that technical advances with URS achieved higher stone-free rates and had fewer complications than previously [21, 22]. However, lower morbidity and economic aspects [2, 23, 24] support the use of SWL, and this explains its continuing prominence in all guidelines. A further explanation is that, based on the published decision tree and consensus tree, the EAU guidelines state that more than 90% of renal and ureteral calculi might be suitable for SWL according to the recent literature [25–27].

One limitation of our study was disregarding hierarchical recommendations when comparing recommended approaches. Since comparing decision trees with multiple weighted recommendations would have resulted in an exuberant consensus tree, we decided to include all recommended therapies for each specific situation and weight them equally to avoid distortion of the comparative analysis through oversimplification. To compensate for this, hierarchical recommendations of different treatment options were analysed separately in our comparative analysis (Table 2). Guideline language is usually restricted to the nation’s main language. Due to linguistic difficulties caused by different font systems or scripts, some national guidelines could not be included in our analysis (e.g. the guidelines of the Japanese Urology Association) or might have remained unnoticed. Moreover, our systematic analysis excluded stone composition, postoperative management, follow-up and specific situations such as staghorn calculi, urolithiasis in pregnancy or in children. Techniques such as laparoscopic and open surgery are part of many guidelines (e.g. SAU, AFU, EAU and AUA). While these approaches are part of the standard treatment recommendations in SAU and AFU guidelines, EAU and AUA guidelines mention the use of these approaches in limited special scenarios only, which is why we did not include the latter in our comparative analysis. Of course, these aspects must be additionally considered in decision-making.

## Conclusion

Six guidelines with recommendations for the surgical treatment of urolithiasis to support urologists in decision-making were available for inclusion in our analysis. While there is generally a high grade of consensus for most stone scenarios, we also detected some relevant discrepancies between different guidelines. In particular, lower consensus was found for the treatment of proximal ureteral stones and hierarchical levels of recommended treatments for specific situations. These are, however, controversial situations where adequate evidence to assist with decision-making has yet to be elicited by further research.
